# Deciphering inhibitory mechanism of coronavirus replication through host miRNAs-RNA-dependent RNA polymerase interactome

**DOI:** 10.3389/fgene.2022.973252

**Published:** 2022-08-26

**Authors:** Olanrewaju B. Morenikeji, Muyiwa S. Adegbaju, Olayinka S. Okoh, Asegunloluwa E. Babalola, Anastasia Grytsay, Olubumi A. Braimah, Mabel O. Akinyemi, Bolaji N. Thomas

**Affiliations:** ^1^ Division of Biological and Health Sciences, University of Pittsburgh at Bradford, Bradford, PA, United States; ^2^ Institute for Plant Biotechnology, Stellenbosch University, Stellenbosch, South Africa; ^3^ Department of Chemical Sciences, Anchor University, Lagos, Nigeria; ^4^ Department of Mathematical Sciences, Anchor University, Lagos, Nigeria; ^5^ Department of Biological Sciences, Fairleigh Dickinson University, Madison, NJ, United States; ^6^ Department of Biomedical Sciences, Rochester Institute of Technology, Rochester, NY, United States

**Keywords:** miRNA, RNA-dependent RNA polymerase, prediction, markers, regulation, coronavirus

## Abstract

Despite what we know so far, Covid-19, caused by SARS-CoV-2 virus, remains a pandemic that still require urgent healthcare intervention. The frequent mutations of the SARS-CoV-2 virus has rendered disease control with vaccines and antiviral drugs quite challenging, with newer variants surfacing constantly. There is therefore the need for newer, effective and efficacious drugs against coronaviruses. Considering the central role of RNA dependent, RNA polymerase (RdRp) as an enzyme necessary for the virus life cycle and its conservation among coronaviruses, we investigated potential host miRNAs that can be employed as broad-range antiviral drugs averse to coronaviruses, with particular emphasis on BCoV, MERS-CoV, SARS-CoV and SARS-CoV-2. miRNAs are small molecules capable of binding mRNA and regulate expression at transcriptional or translational levels. Our hypothesis is that host miRNAs have the potential of blocking coronavirus replication through miRNA-RdRp mRNA interaction. To investigate this, we retrieved the open reading frame (ORF1ab) nucleotide sequences and used them to interrogate miRNA databases for miRNAs that can bind them. We employed various bioinformatics tools to predict and identify the most effective host miRNAs. In all, we found 27 miRNAs that target RdRp mRNA sequence of multiple coronaviruses, of which three - hsa-miR-1283, hsa-miR-579-3p, and hsa-miR-664b-3p target BCoV, SARS-CoV and SARS-CoV-2. Additionally, hsa-miR-374a-5p has three bovine miRNA homologs viz bta-miR-374a, bta-miR-374b, and bta-miR-374c. Inhibiting the expression of RdRp enzyme *via* non-coding RNA is novel and of great therapeutic importance in the control of coronavirus replication, and could serve as a broad-spectrum antiviral, with hsa-miR-1283, hsa-miR-579-3p, and hsa-miR-664b-3p as highly promising.

## Introduction

**FIGURE 1:**
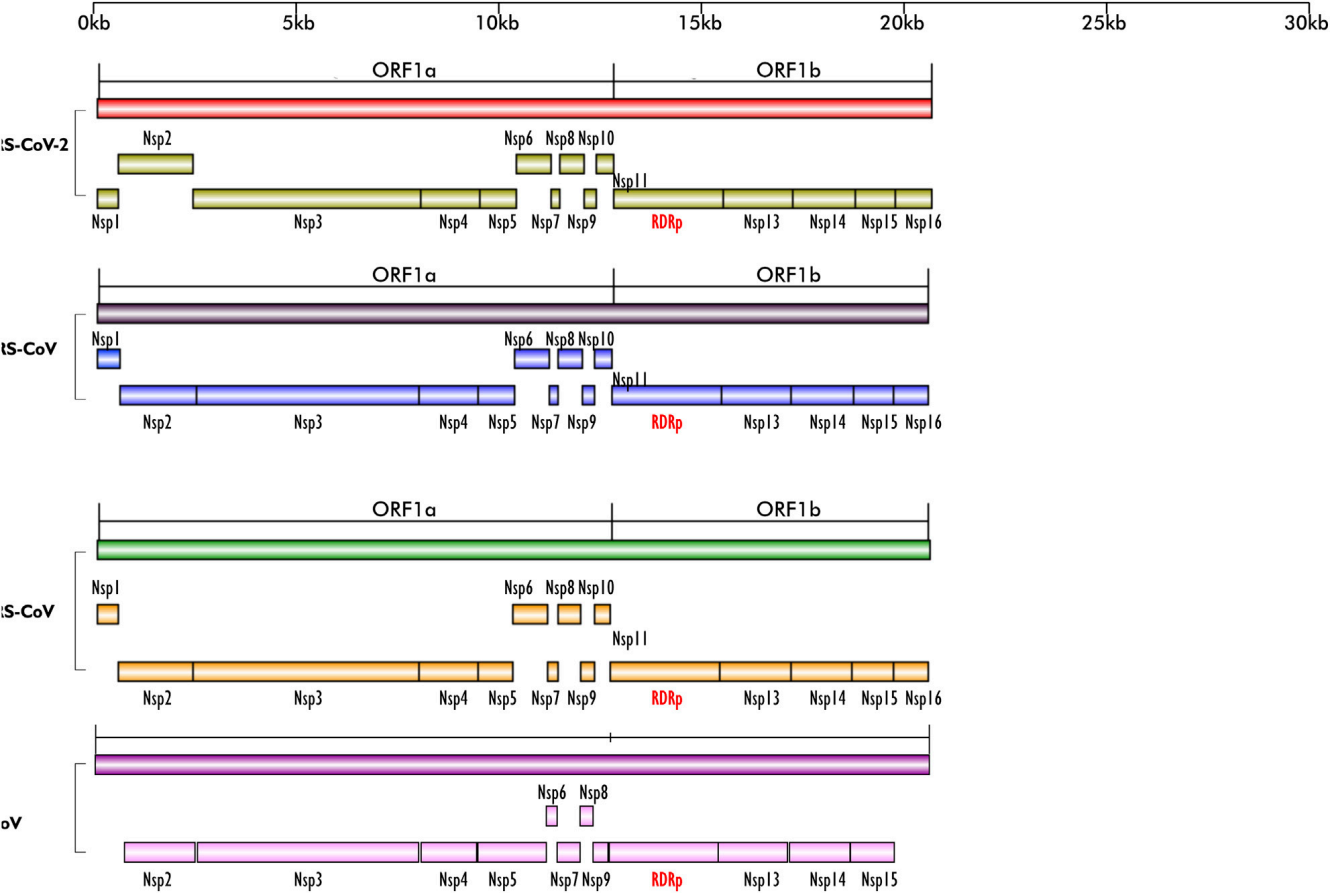
Schematics of the ORF1a and ORF1b regions of the genomes of SARS-CoV-2, MERS-CoV, SARS-CoV and BCoV; their encoded non-structural proteins (nsp) and RdRp layered on one another for easy comparison. The region is highly conserved in the four viruses; except for BCoV which does not have nsp1. All the nsps are present in the four viruses and they are arranged in the same sequence/order. Figures created with sketch pad.

The diseases caused by SARS-CoV-2, a member of the Coronaviridae family, have had profound impact on all human endeavors, leaving hardship, death and destruction in its trail (Aftab et al., 2020). The rate of transmission of SARS-CoV-2 from person to person is the major driver of the significant morbidity and mortality attendant to Covid-19 and its pandemic form (Gao et al., 2020; Walls et al., 2020). After a successful entry into the host, viral replication is another important step to its pathogenicity and transmission. A large portion of coronavirus genome encodes open reading frame (ORF) 1a/1b (Figure 1), which produces two precursor polyproteins (pp1a) and (pp1ab), dedicated to code for multiple enzymes among which is RNA dependent, RNA polymerase (RdRp). Each of these precursor polyproteins are subsequently cleaved into non-structural proteins (nsp). The pp1ab is cleaved into 16 nsps, of which nsp12 or RNA dependent, RNA polymerase (RdRp) is one, and pivotal for successful virus replication in the host (Gao et al., 2020). In addition, formation of protein complex between RdRp protein, nsp seven and nsp eight have been reported, as the latter duo serve as cofactor for RdRp (Kirchdoerfer and Ward, 2019; Gao et al., 2020). Except for retroviruses, most RNA viruses require the activity of RdRp protein for viral replication and may explain why its active site is the most conserved among these viruses (Aftab et al., 2020), thereby making it a prominent target for drug development.

**FIGURE 2:**
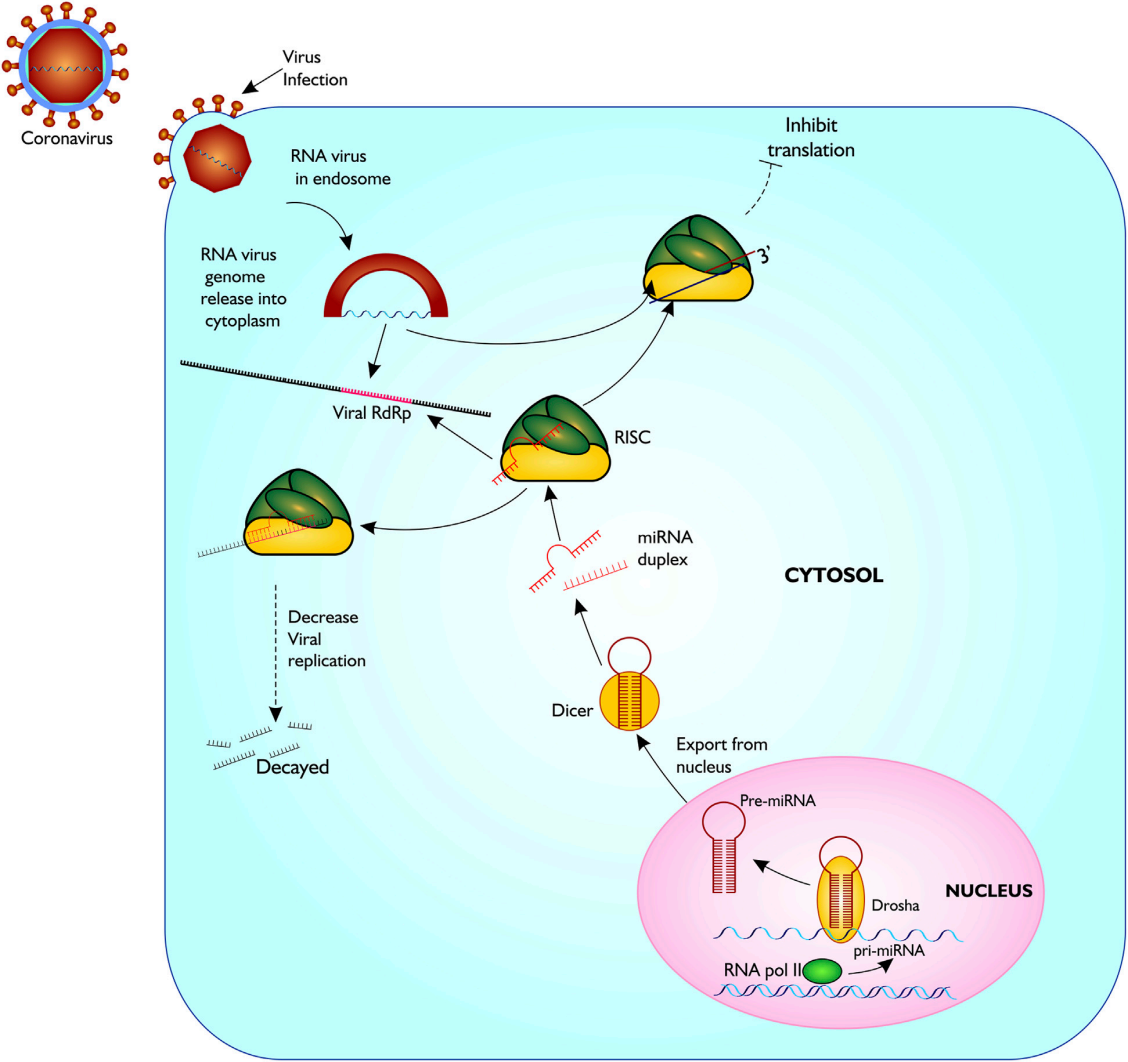
Proposed model of miRNA biogenesis and base pairing with coronavirus RdRp mRNA sequence. The figure gives a description of coronavirus infection on host cell, and release of host miRNA to base pair and degrade the virus or inhibit translation. Figure created with sketch pad.

Several vaccines have now been developed and approved for use to limit COVID-19 infection in humans. However, their safety and long-term efficacy against SARS-CoV-2 is not guaranteed (Saha et al., 2021). Other strategies recommended for treating disease include inhibition of RdRp activity using antiviral agents like the nucleoside analogues, Favipiravir, Galidesivir, and Remdesivir, and other plant-based compounds such as Tellimagrandin I, Saikosaponin B2, Hesperidin and Epigallocatechin gallate (Saha et al., 2021). So far, these antiviral drugs have been reported to be ineffective against SARS-CoV-2, possibly due to single nucleotide polymorphism (SNP)-induced changes culminating in conformational, structural and functional amino acids changes and the high virus mutation rate. Therefore, alternate therapeutic options that are effective against the virus must be explored. Here, we propose an alternative option that utilizes blocking RdRp transcript *via* host microRNAs thereby inhibiting translation of the most important protein for viral replication, leading to reduced viral propagation and pathogenicity (Figure 2).

MicroRNAs are short non-coding RNAs, of about 23 nucleotides transcribe by RNA polymerase II from the genome of an organism (Trobaugh and Klimstra, 2017). They control several cellular operations at pre- and posttranscription by taking on target transcripts such as host mRNA and RNAs from the genome of pathogens, *via* sequence-specific interlink, influencing the function and/or stability of these targets (Morenikeji et al., 2020; Tucker et al., 2021). Several studies have shown the involvement of miRNAs in regulation of host immune responses. The use of machine learning and bioinformatic tools is pivotal in understanding pre- and post- translational regulation of genes and many biological processes (Bao et al., 2018). Morenikeji et al. (2020) demonstrated *via in silico* analysis that certain bovine miRNAs are involved in regulating specific immune response genes associated with Bovine coronavirus (BCoV) infection and were identified as drug targets and diagnostic biomarker for the virus. Additionally, miRNAs such as gga-miR-1603 and gga-miR-1794 were found in chicken binding the L gene region of Newcastle Disease virus, causing viral degradation and inhibiting replication *in vitro* (Chen et al., 2021). In humans, decrease in viral replication, translation and transmission from person to person due to binding of certain miRNAs to the genome of viruses such as Human immunodeficiency virus (HIV), Enterovirus 71 (E 71) and Human T cell leukemia virus, type I (HTLV-1) have also been reported (Nathans et al., 2009; Zheng et al., 2013; Bai and Nicot 2015). More evidence on the involvement of miRNA in altering viral replication and pathogenesis have continued to emerge (Ingle et al., 2015; Khongnomnan et al., 2015; Trobaugh and Klimstra, 2017), but none of these studies have examined the role of miRNA in inhibiting coronavirus replication through RdRp nucleotide sequence, showing the importance of our study.

Considering the significant role of RdRp in viral replication and survival, we elucidated host miRNAs that can bind mRNA of RdRp in four coronaviruses, resulting in its disintegration, thereby controlling the replication and pathogenesis of RNA viruses and opening a new door to therapeutic targets for coronaviruses.

## Materials and methods

### Sequence mining of RNA-dependent RNA polymerase region from the genome of various coronaviruses

**FIGURE 3:**
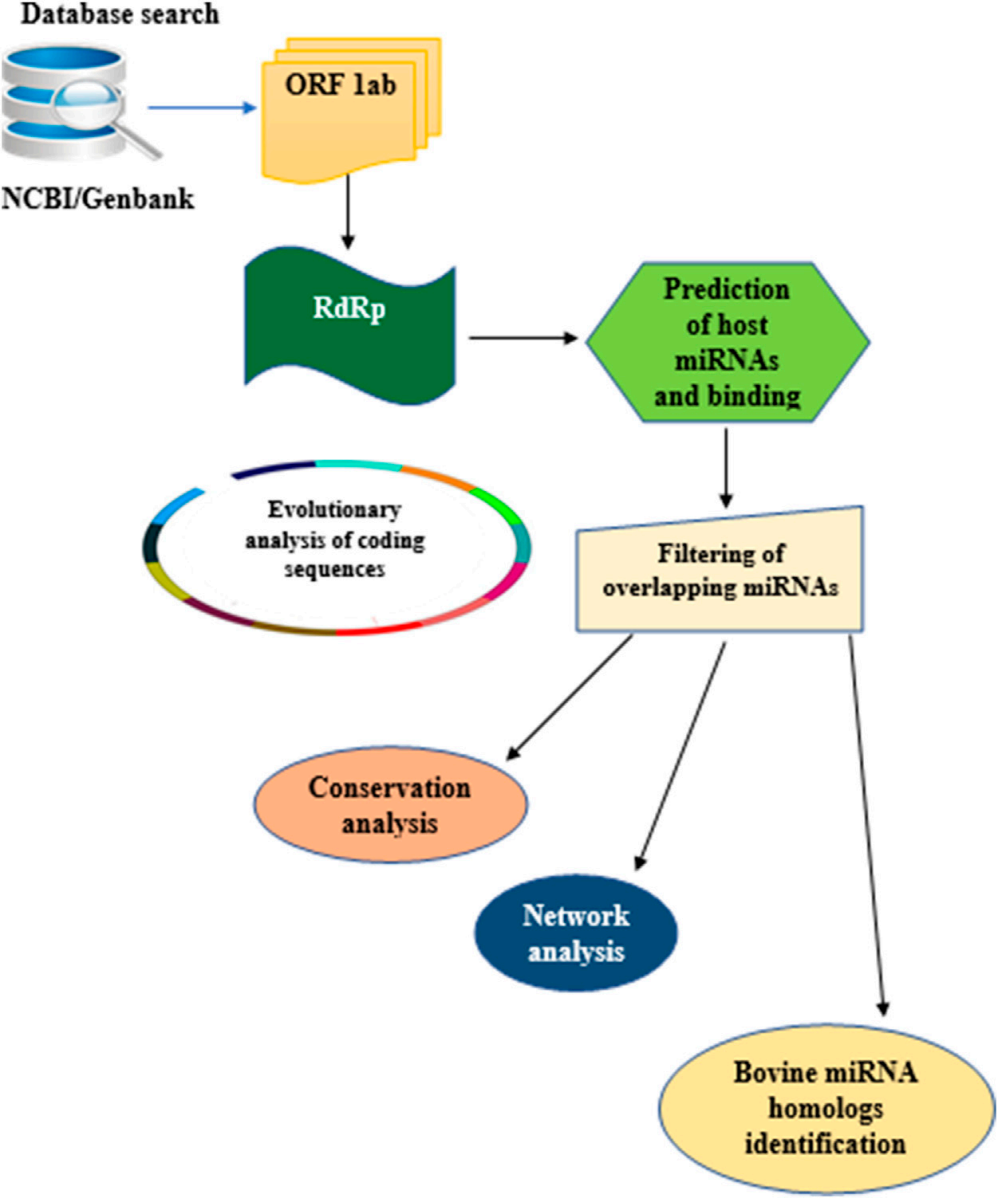
Flow chart of methodology used for the study. Step by Step pipeline for elucidating host miRNA–viral RdRp interaction.

In this study, the analytical pipeline employed, starting from sequence curation to interactome networks, is a slight modification of our previously described model (Figure 3), (Morenikeji et al., 2020, 2021; Tucker et al., 2021). Since RdRp is one of the 16 non-structural proteins encoded by ORF1ab gene of coronaviruses, we carried out an extensive search for ORF1ab gene of 13 selected coronaviruses, whose genomes have either been fully or partially annotated. The corresponding nucleotide sequences and accession numbers were retrieved from NCBI GenBank (https://www.ncbi.nlm.nih.gov/genbank/). These viruses are: SARS-CoV (NC_004718.3), SARS-CoV-2 (NC_045512.2), tylonycteris bat coronavirus (NC_009019.1), MERS-CoV (NC_019843.3), duck coronavirus (NC_048214.1), Canada goose coronavirus (NC_046965.1), BCoV (NC_003045.1), betacoronavirus England 1 (NC_038294.1), alphacoronavirus (NC_046964.1), bat coronavirus (NC_034440.1), pipistrellus bat (NC_009020.1), rabbit coronavirus (NC_017083.1), rodent and coronavirus (NC_046954.1).

### Evolutionary analysis of RNA-dependent RNA polymerase in 13 coronaviruses

To determine the evolutionary relationship and distance of the RdRp region from the 13 coronaviruses, we constructed a phylogenetic tree using https://ngphylogeny.fr/ with the following workflow. Preliminary multiple sequence alignment (MSA) was generated using MAFFT, followed by trimming of the MSA to focus on the informative regions using block mapping and gathering with entropy (BMGE) (Criscuolo and Gribaldo, 2010). The phylogenetic tree was inferred using PhyML (Guindon et al., 2010) and tree visualization carried out with interactive tree of life (iTol) (https://itol.embl.de). Using the MSA generated by BMGE, pairwise distance between the RdRp of the 13 coronaviruses was computed using MEGA X (Kumar et al., 2018).

### Prediction and network of miRNA binding sites in the RNA-dependent RNA polymerase region of BCoV, MERS-CoV, SARS-CoV and SARS-CoV-2

To examine whether host cellular miRNA can target coronavirus RdRp, we selected four common coronaviruses in human and cattle for further analysis. Potential miRNA binding sites in the RdRp nucleotide sequences of BCoV, MERS-CoV, SAR-CoV and SARS-Cov-2 were predicted using mirDB software (http://mirdb.org). Each of the RdRp sequences from the four coronaviruses were used as the target sequence with human genome selected as the reference for miRNA prediction. After each prediction, miRNAs with a score of 60 and above were considered significant and selected for further analysis. The list of miRNAs from each coronavirus were intersected with Bioinformatic and Evolutionary Genomics (BEG) Venn diagram generator (http://bioinformatics.psb.ugent.be/webtools/Venn/). Based on the complementary base pairing of miRNAs and RdRp mRNA and the value of normalized binding free energy (ndG), possible miRNA-mRNA interactome network connections were determined using Cytoscape version 3.7.2, as previously described (Morenikeji et al., 2020; Tucker et al., 2021). To search for possible homologs of human miRNAs in the bovine genome, we searched the miRNA database (https://mirbase.org). The sequence of each of the top 25 miRNAs selected were used as query against the *Bos taurus* genome on mirDB (http://mirdb.org). Homologous bovine miRNAs were extracted and recorded for further analysis.

## Results

### Dataset of RNA-dependent RNA polymerase nucleotides from the genome of 13 coronaviruses

Our search for nucleotide sequences encoding RdRp in coronaviruses using the keyword “1 ab polyprotein” initially yielded about 59 organisms. After filtering for only coronaviruses, 13 viruses, whose genomes were either fully or partially annotated were subsequently selected for further analyses. The sequences encoding RdRp regions of tylonycteris bat coronavirus, MERS-CoV, duck coronavirus, SARS-CoV, Canada goose coronavirus, BCoV, betacoronavirus England 1, alphacoronavirus, bat coronavirus, SARS-CoV-2, pipistrellus bat, rabbit coronavirus and rodent coronavirus were identified to be within the ORF1ab gene (Table 1). Since RdRp protein is categorized to be one of the cleaved 16 non-structural proteins encoded by ORF1ab gene, its coding region which falls between nsp11 and nsp 13, is beyond the coding region of ORF1a gene, which partially overlaps with ORF1ab gene and encodes variants of nsp1 to nsp9 (Figure 1). Each of the 13 coronaviruses’ RdRp nucleotide sequences were extracted and added to the pipeline (Figure 3) to determine their evolutionary relationship.

**TABLE 1:** List of coronavirus species; accession number of their ORF1ab gene, genome location and the location of RdRp coding sequence within ORF1ab genome location.

S/N	Virus	Accession number	Genome location of ORF1ab	Location of RdRp within ORF1ab
1	Tylonycteris bat CoV	NC_009019.1	267–21625	13553–16327
2	SARS-CoV	NC_004718.3	265..21485	13401–16163
3	MERS-CoV	NC_019843.3	279–21514	13410–16207
4	Duck coronavirus	NC_048214.1	347..20364	12211–15071
5	Bovine coronavirus	NC_003045.1	211..21494	13318–16100
6	Canada goose CoV	NC_046965.1	554..20085	11971–14786
7	Betacoronavirus England 1	NC_038294.1	278–21513	13400–16185
8	Alphacoronavirus Bat-CoV	NC_046964.1	281–20175	12136–14885
9	Bat CoV	NC_034440.1		13156–15970
10	Pipistrellus bat coronavirus	NC_009020.1	261–21808	13661–16332
11	Rabbit coronavirus	NC_017083.1	209–21663	13483–16270
12	Rodent coronavirus	NC_046954.1	211–21596	13386–16201
13	SARS-CoV-2	NC_045512.2	266–21555	13430–16221

### Evolutionary relatedness of RNA-dependent RNA polymerase coding sequences

**FIGURE 4:**
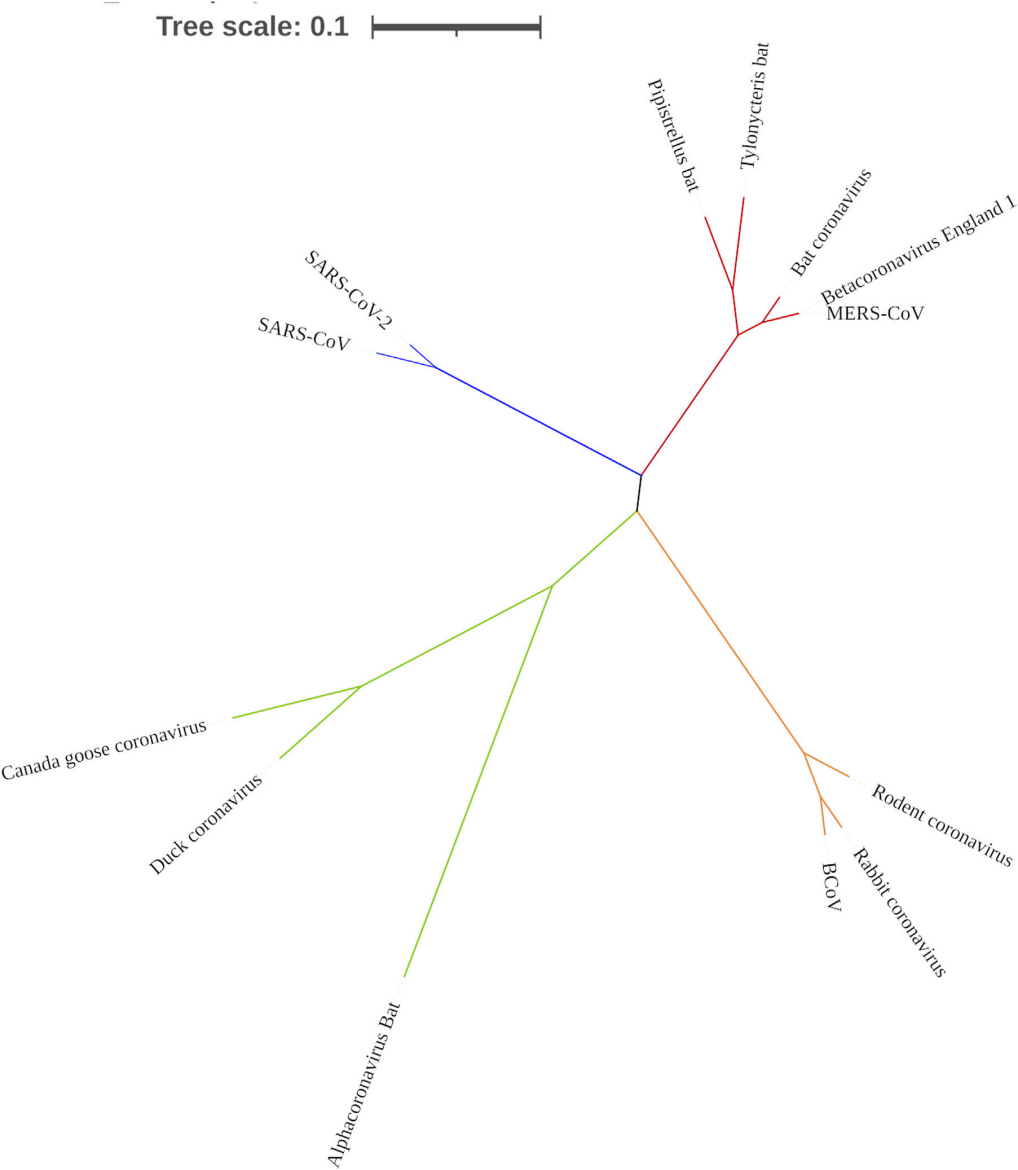
Phylogenetic tree showing the evolutionary relationship of 13 coronaviruses. Tree was constructed using MEGA X.

To confirm that RdRp coding sequence is conserved among the coronaviruses, we examined their evolutionary relatedness through pairwise distance and phylogenetic analysis. The nucleotide sequence analysis of RdRp reveals minor variation across the 13 viruses though sharing common evolutionary origin (Figure 4). Two viruses, MERS-CoV and Betacoronavirus England 1, are not different from each other in this region, showing a pairwise distance of 0.00 (Table 2). Similarly, comparing the RdRp sequences of bat coronavirus with either MERS-CoV or Betacoronavirus England one indicated some level of closeness with a value of 0.18. A similar close relatedness was observed for SARS-CoV and SARS-CoV-2 having a pairwise distance of 0.29. Alphacoronavirus is the most distantly related from the rest of the viruses, showing consistent higher value for the pairwise distance, further supported by the phylogenetic tree analysis (Figure 4).

**TABLE 2:** Genetic pairwise distance of the 13 coronaviruses used in the study.

	Tylonycteris bat coronavirus	SARS- CoV	MERS CoV	Duck coronavirus	Canada goose coronavirus	B-CoV	Beta- Coronavirus england 1	Alpha- Coronavirus bat	Bat coronavirus	Pipistrellus bat coronavirus	Rabbit coronavirus	Rodent coronavirus	SARS-CoV-2
Tylonycteris bat coronavirus													
SARS-Cov	4.36												
MERS-CoV	1.8	4.25											
Duck coronavirus	4.85	5.27	4.83										
Canada goose coronavirus	4.89	5.17	4.81	2.34									
B-CoV	4.48	4.92	4.52	5.28	5.14								
Beta-coronavirus England 1	1.8	4.27	0	4.86	4.84	4.55							
Alpha-coronavirus bat	5.1	5.32	5.13	4.99	5.11	5.51	5.13						
Bat coronavirus	1.75	4.33	0.18	4.76	4.93	4.58	0.18	4.98					
Pipistrellus bat coronavirus	1.57	4.43	1.56	5.11	5.14	4.61	1.62	5.41	1.54				
Rabbit coronavirus	4.49	4.95	4.57	5.26	5.24	0.2	4.6	5.51	4.59	4.63			
Rodent coronavirus	4.57	4.83	4.58	5.26	5.21	0.57	4.61	5.44	4.56	4.71	0.54		
SARS-Cov-2	4.27	0.29	3.97	5.21	5.07	4.8	3.99	5.27	4.21	4.34	4.73	4.65	

The least distance is 0; which is between MERS-CoV and Beta coronavirus England one; while 5.51 is the highest pairwise genetic distance and this is between BCoV and Alpha coronavirus bat; and between Alpha-coronavirus bat and rabbit coronavirus.

### Open reading frame 1ab is conserved in BCoV, MERS-CoV, SARS-CoV and SARS-CoV-2

From the genome organization of BCoV, MERS-CoV, SARS-CoV and SARS-CoV-2 (Figure 1), ORF1ab in the viruses are very similar, indicating high level of conservation in this gene making it an excellent antiviral drug candidate. To ascertain the degree of conservation in BCoV, MERS-CoV, SARS-CoV and SARS-CoV-2, the well annotated ORF1ab genes of each of the viruses were overlayed on one another and compared. Comparing the gene across the viruses, we found that ORF1ab is highly conserved across the viruses as no conspicuous difference was noted in the arrangement of all the non-structural proteins and RdRp (Figure 1), emphasizing the choice of this region of RdRp as an excellent potential antiviral drug target.

### Identification of miRNA binding to RNA-dependent RNA polymerase of BCoV, MERS-CoV, SARS-CoV and SARS-CoV-2

**FIGURE 5:**
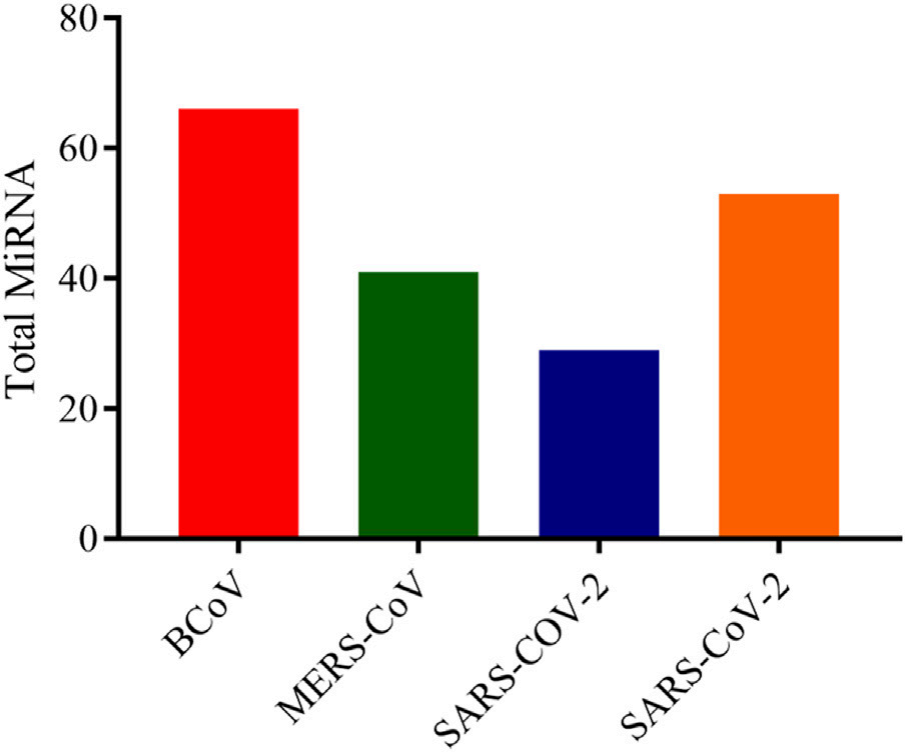
Bar chart showing the number of predicted human miRNA that can bind with the ORF1ab region of each of BCoV, MERS-CoV, SARS-CoV and SARS-CoV-2. Figure created with graphpad.

**FIGURE 6:**
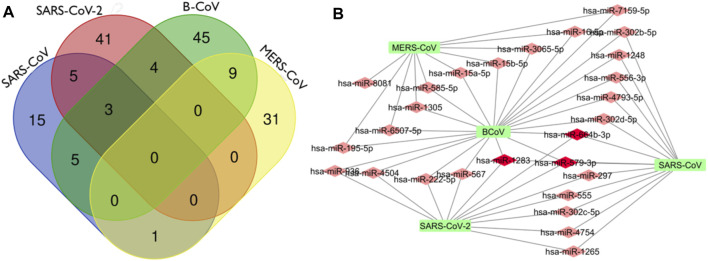
Venn Diagram (http://bioinformatics.psb.ugent.be/webtools/Venn/) showing the number of predicted human miRNA that can target multiple coronaviruses. The number in the intersection/overlapping regions represent the number of miRNAs that can concomitantly target the coronaviruses represented by the intersected shape **(A)**. Network connections among miRNAs and RdRp of SARS_CoV, B_CoV, MERS_CoV and SARS_CoV-2. **(B)** Generated using Cytoscape 3.7.2.

In this analysis, BCoV, MERS-CoV, SARS-CoV and SARS-CoV-2 miRNAs that bind to the RNA-dependent RNA polymerase (RdRp) of coronaviruses were examined. A total of one hundred and three (103) miRNAs were obtained for BCoV, seventy-eight (78) for MERS-CoV, fifty-seven (57) for SARS-CoV, and ninety-seven (97) for SARS-CoV-2. To ensure the binding of miRNAs to RdRp target, significant miRNAs were filtered based on the ranking scores as described above. The filtering generated a total of sixty-six (66) miRNAs for BCoV, forty-one (41) for MERS-CoV, twenty-nine (29) for SARS-CoV and fifty-three (53) for SARS-CoV-2 (Figure 5). The results of complementary binding of human miRNAs to the RdRp sequence for each of the four coronaviruses were intersected to identify broad-spectrum miRNAs, which can possibly inhibit viral replication. As shown, there was no miRNA that concomitantly bind to the RdRp region of all four viruses (Figure 6A). However, we uncovered three miRNAs; hsa-miR-1283, hsa-miR-664b-3p and hsa-miR-579-3p that could bind to this region in BCoV, SARS-CoV and SARS-CoV-2 (Table 3; Figure 6B). Similarly, miRNAs that could bind to the region in at least two coronaviruses were identified, ranging from as low as one (hsa-miR-8081) for MERS-CoV and SARS-CoV to as high as nine (hsa-miR-585-5p, hsa-miR-7159-5p, hsa-miR-1305, hsa-miR-15a-5p, hsa-miR-6507-5p, hsa-miR-16-5p, hsa-miR-3065-5p, hsa-miR-195-5p and hsa-miR-15b-5p) for BCoV and MERS-CoV (Table 3).

**TABLE 3:** Predicted miRNAs with regions of complementarity in multiple coronaviruses from BCoV, MERS-CoV, SARS-CoV and SARS-CoV-2.

RdRp from virus	Number of intercepts	miRNA
|B_Cov | SARS_Cov | SARS_Cov2 |	3	hsa-miR-1283 hsa-miR-579-3p hsa-miR-664b-3p
| SARS_Cov | SARS_Cov2 |	5	hsa-miR-4754 hsa-miR-555 hsa-miR-297 hsa-miR-1265 hsa-miR-302c-5p
|B_Cov | SARS_Cov |	5	hsa-miR-4793-5p hsa-miR-302b-5p hsa-miR-556-3p hsa-miR-1248 hsa-miR-302d-5p
| MERS_CoV | SARS_Cov |	1	hsa-miR-8081
B_Cov SARS_Cov2	4	hsa-miR-4504 hsa-miR-222-5p hsa-miR-567 hsa-miR-936
B_Cov MERS_CoV	9	hsa-miR-7159-5p hsa-miR-585-5p hsa-miR-1305 hsa-miR-195-5p hsa-miR-6507-5p hsa-miR-3065-5p hsa-miR-15a-5p hsa-miR-15b-5p hsa-miR-16-5p

Number of intercepts show the number of miRNAs, with complementary region.

Interestingly, there was no miRNA concomitantly binding RdRp region in both MERS-CoV and SARS-CoV-2 (Figure 6A). The identity of the connections of miRNAs and RdRp between four coronaviruses (BCoV, MERS-CoV, SARS-CoV and SARS-CoV-2) are depicted through a network as shown (Figure 6B). This interactome reveals a possible molecular mechanism for regulating multiple coronavirus replication through miRNAs binding RdRp mRNA. Network of different nodes were created based on all identified miRNAs and potential binding sites on RdRp mRNA, while the network edges were determined through the value ndGs and correlation between each RNA. From Table 3, it is shown that 27 human miRNAs targeted multiple viruses from BCoV, MERS-CoV, SARS-CoV and SARS-CoV-2. Of particular importance, among these 27 miRNAs are three miRNAs (hsa-miR-1283, hsa-miR-579-3p and hsa-miR-664b-3p) that are predicted to target BCoV, SARS-CoV and SARS-CoV-2. Additionally, we report five miRNAs targeting SARS-CoV and SARS-CoV-2, while another five targeted BCoV and SARS-CoV.

### Human miRNA homologs found in bovine genome target RNA-dependent RNA polymerase mRNA sequences

Of the top 25 human miRNAs selected for further analysis, eight has bovine miRNA homologs as shown (Table 4). Interestingly, one of the human miRNAs, hsa-miR-374a-5p, had three bovine miRNA homologs including bta-miR-374a, bta-miR-374b, and bta-miR-374c. hsa-miR-3065-5p has two bovine homologs - bta-miR-338 and bta-miR-3065, while others have one homolog each. In all, we report 13 bovine homologs, and two of them, bta-miR-196a and bta-miR-338 are read in reverse direction while eleven are forward stranded.

**TABLE 4:** Predicted human miRNAs that have bovine miRNA homologs, their size and strands.

miRNA	Seed location	Bovine homolog	Size	Strand
hsa-miR-196a-1-3p	229, 1027, 1413, 1765, 1940	bta-miR-196a	3 to 20	-
hsa-miR-654-5p	1387	bta-miR-380-5p	1 to 22	+
hsa-miR-541-3p	1387	bta-miR-541	1 to 22	+
hsa-miR-374a-5p	1047, 1362, 1370	bta-miR-374a	1 to 22	+
		bta-miR-374b	2 to 22	+
		bta-miR-373c	1 to 21	+
hsa-miR-664b-3p	1628, 2626	bta-miR-664b	3 to 23	+
hsa-miR-545-5p	830, 2750	bta-miR-545-5p	1 to 21	+
hsa-miR-374b-5p	1047, 1362, 1370	bta-miR-374b	1 to 22	+
		bta-miR-374a	1 to 22	+
		bta-miR-374c	1 to 21	+
hsa-miR-3065-5p	227, 512, 1025	bta-miR-338	1 to 23	-
		bta-miR-3065	1 to 23	+

The seed location is with respect to the human miRNA, while the size and strand are the bovine miRNAs—strand miRNAs are read in reverse direction.

## Discussion

The genome arrangement of coronaviruses is similar and of particular importance is the open reading frame 1 ab (ORF1ab) gene, which encodes 1 ab polyprotein, a protein precursor that is further cleaved into sixteen non-structural proteins (nsps). One of the sixteen nsps is RdRp that plays a vital role in RNA virus replication (Aftab et al., 2020; Gao et al., 2020; Jiang et al., 2021). RdRp protein is a promising candidate for drug target for treating diseases caused by coronavirus because the active site is highly conserved and the protein lacks homologous counterparts in host cell (Jiang et al., 2021). Medical interventions in form of mRNA vaccines and antiviral drugs have been developed and approved to treat coronavirus disease such as Covid-19, but many of these drugs are still undergoing clinical trials. The concept behind antiviral drugs for treating Covid-19 and other diseases caused by RNA viruses is to identify compounds which can bind to active site of the RdRp enzymes and prevent its catalytic activity, which leads to viral replication (Markland et al., 2000; Yang et al., 2011; Elfiky 2016; Elfiky et al., 2017; Elfiky and Ismail 2019; Ezat et al., 2019; Wang et al., 2020). However, major concern on the efficacy of the antiviral drugs remains, necessitating exploring alternative options, including preventing RdRp protein translation. To our knowledge, the use of non-coding RNA such as miRNAs as an alternative route has not been explored as antiviral drug option. Since miRNA can bind directly to the genome of RNA virus or cause changes in the host transcriptome facilitated by the virus, it is noteworthy that finding host miRNA that can bind directly to the RdRp region of coronaviruses may provide insight on effective manipulation to control viral replication/load in the host and provide a remarkable alternative treatment.

To have an effective antiviral drug, it is important that a virus target be conserved. Therefore, we identify the conservation of miRNA binding site in the RdRp sequence of multiple coronaviruses through evolutionary analysis. First, 13 annotated RdRp nucleotide sequences were used to define the conservation of this region and construct a phylogenetic tree. Most of the viral species examined belong to the betacoronavirus subfamily, with our result showing high similarity between them, revealing this region as a potential drug target. Interestingly, the pairwise distance between MERS-CoV and Betacoronavirus England one show no difference in this region for both viruses, indicating that they are likely to have the same binding site for host miRNAs. A close similarity in the RdRp region of SARS-CoV and SARS-CoV-2 shows the virus evolved from a common origin, in agreement with previous findings (Aftab et al., 2020; Wu et al., 2020). The genetic conservation of RdRp gene across multiple viruses shows a strong positive selection for this region and justifies the fact that the enzyme coded by this region is important for almost all RNA virus replication, strengthening its choice for miRNA drug targeting. It is puzzling that the magnitude of the difference in pathogenicity, rate of transmission and virulence between SARS-CoV and SARS-CoV-2 is only caused by single nucleotide mutations (Ceraolo and Giorgi, 2020; Kruse, 2020; Lu et al., 2020; Nguyen et al., 2020; Wang et al., 2020). Thus, the slight difference in the pairwise distance as observed between the RdRp sequences of SARS-CoV and SARS-CoV-2 may have remarkable implications on the number of miRNAs which can bind concomitantly with both viruses. Several regions of RNA viruses mutate at a faster rate as a mechanism to escape host immune system reaction. However, a slower mutation rate at the RdRp region means a miRNA could be broad spectrum antiviral drug for many viruses.

Remarkably, our study uncovered several miRNAs that bound to the RdRp sequence of coronaviruses including BcoV, MERS-CoV, SARS-CoV andSARS-CoV-2. The presence of human miRNA homologs in bovine genome is of great importance as this is indicative of the crucial functional role these miRNAs play has been preserved by evolutionary forces or selection. Additionally, some of the miRNAs have multiple binding sites within the RdRp region thereby increasing the binding probability and reducing off-target effects, strengthening their choice for possible antiviral molecule. This is contrary to the submission of Thorg et al. (2017), which stated that the common location of the miRNA binding site is the untranslated regions of the viral genome. Conversely, our results align with a similar study in chicken that identified multiple miRNA binding sites within the L gene of the NDV and infectious bursal disease virus (IBDV), reporting that the overexpression of ggam-miR-21 inhibits VP1 translation in chicken fibroblasts and suppresses overall viral replication (Chen et al., 2021).

We identified some miRNAs including hsa-miR-1283, hsa-miR-664b-3p, hsa-miR-579-3p, which targeted multiple regions in the RdRp sequence of BCoV, SARS-CoV, and SARS-CoV-2; these miRNAs have been previously linked with onco-protective roles, indicating their growth regulatory function. For example, hsa-miR-1283 has been connected with cardio-protection and inhibition of apoptosis (Liu et al., 2021), thereby blocking oncogenesis. In addition, this miRNA has also been implicated in hypertension (Chen et al., 2021). hsa-miR-579-3p, on the other hand has been reported to be associated with growth control and tumor suppression *via* control of melanoma progression (Fattore et al., 2016; Kalhori et al., 2019), while hsa-miR-664b-3p is reported to play a critical role in regulating cancer progression (Liu et al., 2020). From our study, an upregulation or administration of any of the three miRNAs might play a dual role of blocking viral replication/degradation and inhibition of cancer progression.

## Conclusion

In summary, we utilized several computational approaches to examine genome plasticity and elucidate potential host miRNAs that could bind to the RdRp sequence region of coronaviruses. Although viral genome is known to be variable, we report high conservation of RdRp sequence in multiple coronaviruses, indicating evolutionary favorability, hence a candidate signature for genome targeting. In all, this study also provide an insight into possible alternative route for targeting and inhibiting viral replication *via* host non-coding RNA (miRNAs) to combat disease rather than common anti-coronavirus drug, based on inhibiting RdRp enzymatic activities. In particular, hsa-miR-1283, hsa-miR-664b-3p, hsa-miR-579-3p and hsa-miR-374a-5p with bovine homologs bta-miR-374a, bta-miR-374b, and bta-miR-374c are very promising. This study opens the door for developing non-coding RNAs as a broad-spectrum antiviral therapy and lays a foundation for further investigation to validate the effective binding of identified miRNAs to RdRp sequences of coronaviruses through *in vivo* or *in vitro* analysis.

## Data Availability

The original contributions presented in the study are included in the article/Supplementary Material, further inquiries can be directed to the corresponding author.

## References

[B1] Aftab S. O. Ghouri M. Z. Masood M. U. Haider Z. Khan Z. Ahmad A. (2020). Analysis of SARS-CoV-2 RNA-dependent RNA polymerase as a potential therapeutic drug target using a computational approach. J. Transl. Med. 18 (1), 275. 10.1186/s12967-020-02439-0 32635935 PMC7339606

[B2] Bai X. T. Nicot C. (2015). miR-28-3p is a cellular restriction factor that inhibits human T cell leukemia virus, type 1 (HTLV-1) replication and virus infection. J. Biol. Chem. 290, 5381–5390. 10.1074/jbc.M114.626325 25568327 PMC4342455

[B3] Bao W. Yuan C-A. Zhang Y. Han K. Nandi A. K. Honig B. (2018). Mutli-features prediction of protein translational modification sites. IEEE/ACM Trans. Comput. Biol. Bioinform. 15, 1453–1460. 10.1109/TCBB.2017.2752703 28961121

[B4] Ceraolo C. Giorgi F. M. (2020). Genomic variance of the 2019‐nCoV coronavirus. J. Med. Virol. 92 (5), 522–528. 10.1002/jmv.25700 32027036 PMC7166773

[B5] Chen W. Liu T. Liang Q. Chen X. Tao W. Fang M. (2021). miR-1283 contributes to endoplasmic reticulum stress in the development of hypertension through the activating transcription factor-4 (ATF4)/C/EBP-Homologous protein (CHOP) signaling pathway. Med. Sci. Monit. 27, e930552–1. 10.12659/MSM.930552 33911065 PMC8095088

[B6] Chen Y. Zhu S. Hu J. Hu Z. Liu X. Wang X. (2021). gga-miR-1603 and gga-miR-1794 directly target viral L gene and function as a broad-spectrum antiviral factor against NDV replication. Virulence 12 (1), 45–56. 10.1080/21505594.2020.1864136 33372825 PMC7781659

[B7] Criscuolo A. Gribaldo S. (2010). BMGE (block mapping and gathering with entropy): a new software for selection of phylogenetic informative regions from multiple sequence alignments. BMC Evol. Biol. 10, 210. 10.1186/1471-2148-10-210 20626897 PMC3017758

[B8] Elfiky A. A. Ismail A. (2019). Molecular dynamics and docking reveal the potency of novel GTP derivatives against RNA-dependent RNA polymerase of genotype 4a HCV. Life Sci. 238, 116958. 10.1016/j.lfs.2019.116958 31628915

[B9] Elfiky A. A. Mahdy S. M. Elshemey W. M. (2017). Quantitative structure‐activity relationship and molecular docking revealed a potency of anti‐hepatitis C virus drugs against human corona viruses. J. Med. Virol. 89 (6), 1040–1047. 10.1002/jmv.24736 27864902 PMC7167072

[B10] Elfiky A. A. (2016). Zika viral polymerase inhibition using anti‐HCV drugs both in market and under clinical trials. J. Med. Virol. 88 (12), 2044–2051. 10.1002/jmv.24678 27604059

[B11] Ezat A. A. Elfiky A. A. Elshemey W. M. Saleh N. A. (2019). Novel inhibitors against wild-type and mutated HCV NS3 serine protease: An *in silico* study. VirusDisease 30 (2), 207–213. 10.1007/s13337-019-00516-7 31179358 PMC6531565

[B12] Fattore L. Mancini R. Acunzo M. Romano G. Laganà A. Pisanu M. E. (2016). miR-579-3p controls melanoma progression and resistance to target therapy. Proc. Natl. Acad. Sci. U. S. A. 113 (34), E5005–E5013. 10.1073/pnas.1607753113 27503895 PMC5003278

[B13] Gao Y. Yan L. Huang Y. Liu F. Zhao Y. Cao L. (2020). Structure of the RNA-dependent RNA polymerase from COVID-19 virus. Science 368 (6492), 779–782. 10.1126/science.abb7498 32277040 PMC7164392

[B14] Guindon S. Dufayard J. F. Lefort V. Anisimova M. Hordijk W. Gascuel O. (2010). New algorithms and methods to estimate maximum-likelihood phylogenies: Assessing the performance of PhyML 3.0. Syst. Biol. 59, 307–321. 10.1093/sysbio/syq010 20525638

[B15] Ingle H. Kumar S. Raut A. A. Mishra A. Kulkarni D. D. Kameyama Tet al. (2015). The microRNA miR-485 targets host and influenza virus transcripts to regulate antiviral immunity and restrict viral replication. Sci. Signal. 8 (406), ra126. 10.1126/scisignal.aab3183 26645583

[B16] Jiang Y. Yin W. Xu H. E. (2021). RNA-dependent RNA polymerase: Structure, mechanism, and drug discovery for COVID-19. Biochem. Biophys. Res. Commun. 538, 47–53. 10.1016/j.bbrc.2020.08.116 32943188 PMC7473028

[B17] Kalhori M. R. Irani S. Soleimani M. Arefian E. Kouhkan F. (2019). The effect of miR‐579 on the PI3K/AKT pathway in human glioblastoma PTEN mutant cell lines. J. Cell. Biochem. 120 (10), 16760–16774. 10.1002/jcb.28935 31243804

[B18] Khongnomnan K. Makkoch J. Poomipak W. Poovorawan Y. Payungporn S. (2015). Human miR-3145 inhibits influenza a viruses replication by targeting and silencing viral PB1 gene. Exp. Biol. Med. 240 (12), 1630–1639. 10.1177/1535370215589051 PMC4935342 26080461

[B19] Kirchdoerfer R. N. Ward A. B. (2019). Structure of the SARS-CoV nsp12 polymerase bound to nsp7 and nsp8 co-factors. Nat. Commun. 10, 2342. 10.1038/s41467-019-10280-3 31138817 PMC6538669

[B20] Kruse R. L. (2020). Therapeutic strategies in an outbreak scenario to treat the novel coronavirus originating in Wuhan, China. F1000Res. 9, 72. 10.12688/f1000research.22211.2 32117569 PMC7029759

[B21] Kumar S. Stecher G. Li M. Knyaz C. Tamura K. (2018). Mega X: Molecular evolutionary genetics analysis across computing platforms. Mol. Biol. Evol. 35, 1547–1549. 10.1093/molbev/msy096 29722887 PMC5967553

[B22] Liu C. Liu H. Sun Q. Zhang P. (2021). MicroRNA 1283 alleviates cardiomyocyte damage caused by hypoxia/reoxygenation via targeting GADD45A and inactivating the JNK and p38 MAPK signaling pathways. Kardiol. Pol. 79 (2), 147–155. 10.33963/KP.15696 33293495

[B23] Liu T. Meng W. Cao H. Chi W. Zhao L. Cui W. (2020). lncRNA RASSF8-AS1 suppresses the progression of laryngeal squamous cell carcinoma via targeting the miR-664b-3p/TLE1 axis. Oncol. Rep. 44 (5), 2031–2044. 10.3892/or.2020.7771 33000257 PMC7551431

[B24] Lu R. Zhao X. Li J. Niu P. Yang B. Wu H. (2020). Genomic characterisation and epidemiology of 2019 novel coronavirus: Implications for virus origins and receptor binding. Lancet 395 (10224), 565–574. 10.1016/S0140-6736(20)30251-8 32007145 PMC7159086

[B25] Markland W. McQuaid T. J. Jain J. Kwong A. D. (2000). Broad-spectrum antiviral activity of the IMP dehydrogenase inhibitor VX-497: A comparison with ribavirin and demonstration of antiviral additivity with alpha interferon. Antimicrob. Agents Chemother. 44 (4), 859–866. 10.1128/AAC.44.4.859-866.2000 10722482 PMC89783

[B26] Morenikeji O. B. Bernard K. Strutton E. Wallace M. Thomas B. N. (2021). Evolutionarily conserved long non-coding RNA regulates gene expression in cytokine storm during COVID-19. Front. Bioeng. Biotechnol. 8. 10.3389/fbioe.2020.582953 PMC7844208 33520952

[B27] Morenikeji O. B. Wallace M. Strutton E. Bernard K. Thomas B. N. (2020). Integrative network analysis of predicted miRNA-targets regulating expression of immune response genes in bovine coronavirus infection. Front. Genet. 11, 584392. 10.3389/fgene.2020.584392 33193717 PMC7554596

[B28] Nathans R. Chu C. Y. Serquina A. K. Lu C. C. Cao H. Rana T. M. (2009). Cellular microRNA and P bodies modulate host-HIV-1 interactions. Mol. Cell 34, 696–709. 10.1016/j.molcel.2009.06.003 19560422 PMC2763548

[B29] Nguyen T. M. Zhang Y. Pandolfi P. P. (2020). Virus against virus: a potential treatment for 2019-nCov (SARS-CoV-2) and other RNA viruses. Cell Res. 30 (3), 189–190. 10.1038/s41422-020-0290-0 32071427 PMC7054296

[B30] Saha S. Nandi R. Vishwakarma P. Prakash A. Kumar D. (2021). Discovering potential RNA dependent RNA polymerase inhibitors as prospective drugs against COVID-19: an *in silico* approach. Front. Pharmacol. 12, 634047. 10.3389/fphar.2021.634047 33716752 PMC7952625

[B31] tenOever B, R. (2013). RNA viruses and the host microRNA machinery. Nat. Rev. Microbiol. 11, 169–180. 10.1038/nrmicro2971 23411862

[B32] Trobaugh D. W. Klimstra W. B. (2017). MicroRNA regulation of RNA virus replication and pathogenesis. Trends Mol. Med. 23 (1), 80–93. 10.1016/j.molmed.2016.11.003 27989642 PMC5836316

[B33] Tucker A. R. Salazar N. A. Ayoola A. O. Memili E. Thomas B. N. Morenikeji O. B. (2021). Regulatory network of miRNA, lncRNA, transcription factor and target immune response genes in bovine mastitis. Sci. Rep. 11 (1), 21899. 10.1038/s41598-021-01280-9 34753991 PMC8578396

[B34] Walls A. C. Park Y. J. Tortorici M. A. Wall A. McGuire A. T. Veesler D. (2020). Structure, function, and antigenicity of the SARS-CoV-2 spike glycoprotein. Cell 181 (2), 281–292. e6. 10.1016/j.cell.2020.02.058 32155444 PMC7102599

[B35] Wang M. Cao R. Zhang L. Yang X. Liu J. Xu M. (2020). Remdesivir and chloroquine effectively inhibit the recently emerged novel coronavirus (2019-nCoV) *in vitro* . Cell Res. 30 (3), 269–271. 10.1038/s41422-020-0282-0 32020029 PMC7054408

[B36] Wang Y. S. Ouyang W. Pan Q, X. Wang X. L. Xia X. X. Bi Z. W. (2013). Overexpression of microRNA gga-miR-21 in chicken fibroblasts suppresses replication of infectious bursal disease virus through inhibiting VP1 translation. Antivir. Res. 100 (1), 196–201. 10.1016/j.antiviral.2013.08.001 23954191

[B37] Wu A. Peng Y. Huang B. Ding X. Wang X. Niu P. (2020). Genome composition and divergence of the novel coronavirus (2019-nCoV) originating in China. Cell Host Microbe 27 (3), 325–328. 10.1016/j.chom.2020.02.001 32035028 PMC7154514

[B38] Yang P. L. Gao M. Lin K. Liu Q. Villareal V. A. (2011). Anti-HCV drugs in the pipeline. Curr. Opin. Virol. 1 (6), 607–616. 10.1016/j.coviro.2011.10.019 22440918 PMC3775341

[B39] Zheng Z. Ke X. Wang M. He S. Li Q. Zheng C. (2013). Human microRNA hsa-miR-296-5p suppresses enterovirus 71 replication by targeting the viral genome. J. Virol. 87, 5645–5656. 10.1128/JVI.02655-12 23468506 PMC3648165

